# Photoinhibition and photoinhibition-like damage to the photosynthetic apparatus in tobacco leaves induced by *pseudomonas syringae* pv. *Tabaci* under light and dark conditions

**DOI:** 10.1186/s12870-016-0723-6

**Published:** 2016-01-25

**Authors:** Dan-Dan Cheng, Zi-Shan Zhang, Xing-Bin Sun, Min Zhao, Guang-Yu Sun, Wah Soon Chow

**Affiliations:** College of Life Science, Northeast Forestry University, Harbin, 150040 China; State Key Lab of Crop Biology, College of Life Sciences, College of Horticulture Science and Engineering, Shandong Agricultural University, Tai’an, 271018 China; Division of Plant Science, Research School of Biology, College of Medicine, Biology and Environment, The Australian National University, Acton, ACT 2601 Australia

**Keywords:** Biotic stress, *Pseudomonas syringae* pv. *tabaci*, Photosystem I, Photosystem II, *Nicotiana tabacum*

## Abstract

**Background:**

*Pseudomonas syringae* pv. *tabaci* (*Pst*), which is the pathogen responsible for tobacco wildfire disease, has received considerable attention in recent years. The objective of this study was to clarify the responses of photosystem I (PSI) and photosystem II (PSII) to *Pst* infection in tobacco leaves.

**Results:**

The net photosynthetic rate (Pn) and carboxylation efficiency (CE) were inhibited by *Pst* infection. The normalized relative variable fluorescence at the K step (*W*_k_) and the relative variable fluorescence at the J step (*V*_J_) increased while the maximal quantum yield of PSII (*F*_v_/*F*_m_) and the density of *Q*_A_-reducing PSII reaction centers per cross section (RC/CSm) decreased, indicating that the reaction centers, and the donor and acceptor sides of PSII were all severely damaged after *Pst* infection. The PSI activity decreased as the infection progressed. Furthermore, we observed a considerable overall degradation of PsbO, D1, PsaA proteins and an over-accumulation of reactive oxygen species (ROS).

**Conclusions:**

Photoinhibition and photoinhibition-like damage were observed under light and dark conditions, respectively, after *Pst* infection of tobacco leaves. The damage was greater in the dark. ROS over-accumulation was not the primary cause of the photoinhibition and photoinhibition-like damage. The PsbO, D1 and PsaA proteins appear to be the targets during *Pst* infection under light and dark conditions.

**Electronic supplementary material:**

The online version of this article (doi:10.1186/s12870-016-0723-6) contains supplementary material, which is available to authorized users.

## Background

Under natural conditions, in addition to abiotic stresses, plants are exposed to various biotic stresses, including infection by pathogens and attack by herbivorous pests [1, 2]. Biotic stresses decrease crop yields worldwide by an average of 15 % [3]. Compared with the number of studies on plant infections caused by fungi and viruses, there are relatively few regarding plants infected by bacteria [4]. The effects of bacterial pathogens infection depends on the severity and timing of infection, but also on the particular type of bacteria and on genotype-associated host resistance [5, 6]. Bacterial infections strongly affect photosynthesis. In fact, it has been reported that the genes encoding photosynthetic functions are down regulated [7–9] and changes to photosystem II (PSII) proteins occur in *Pseudomonas syringae*-infected plants [10].

*Pseudomonas syringae* are opportunistic bacterial pathogens that can attack a wide variety of plants [11]. There are at least 50 *P. syringae* pathovars based on their host plant specificities and type of disease symptoms [12, 13]. Previous research has revealed that the maximum PSII quantum yield (*F*_v_*/F*_m_), the quantum yield of open PSII traps (*F*_v_’*/F*_m_’), and nonphotochemical quenching (NPQ) were decreased in *Arabidopsis thaliana* leaves infected with *P. syringae* pv*. tomato DC3000* (*Pto*) [14, 15]. Decreases in the actual photochemical efficiency of PSII (Φ_PSII_) and NPQ were also observed in *Pto-*infected *Phaseolus vulgaris* leaves [16]. Additionally, a decrease in NPQ was observed in *P. syringae* pv*. Phaseolicola* (*Pph*)-infected bean plants, while the *F*_v_*/F*_m_ remained stable [17]. Moreover, decreases in Φ_PSII_ and NPQ were detected in *Pph*-infected ‘Canadian Wonder’ *P. vulgaris* leaves [16]. In contrast, a decrease in *F*_v_’*/F*_m_’ and an increase in NPQ were observed in soybean leaves infiltrated with *P. syringae* pv*. glycinea* [8]. As one of the most important pathovars, *P. syringae* pv. *tabaci* (*Pst*) is a hemibiotrophic bacterial pathogen that parasitizes tobacco leaves, causing the formation of brown spots during an infection referred to as wildfire disease [18, 19]. To better understand how to manage *P. syringae* infections, we focused on the tobacco-*Pst* model pathosystem. Although considerable research has recently been completed on the tolerance to *Pst* [20–22] and the photosynthetic performance of plants infected by the other pathovars mentioned above, little information is available on the photosynthetic performance during tobacco-*Pst* interactions.

The D_1_ protein is the core protein of the PSII reaction center. The inhibition of photosynthesis electron transport (PET) from the primary quinone electron acceptor of PSII (*Q*_A_) to the secondary quinone electron acceptor of PSII (*Q*_B_) may consequently be related to the degradation of the D_1_ protein [23]. Similarly, PsbO, the core component of the oxygen evolving complex (OEC), is critical to the functionality of the OEC [24]. Additionally, photosystem I (PSI) photoinhibition is related to the degradation of PsaA [25]. In several studies, dark conditions were simulated using the PET inhibitors 3-(3,4-dichlorophenyl)-1,1-dimethylurea and 2,5-dibromo-3-methyl-6-isopropylbenzoquinone [26, 27]. However, this study focused on PET as influenced by *Pst* infection. Therefore, these inhibitors were not used.

Our objectives were to identify the differences in PSI and PSII responses to light and dark conditions following *Pst* infection of tobacco leaves. We also aimed to determine if photoinhibition occurs during *Pst* infection. To address these questions, we (1) evaluated the changes to the donor and acceptor sides and the reaction center of PSII as well as the PSI activity after *Pst* infection, (2) monitored the production of reactive oxygen species (ROS), and (3) performed Western blot analyses of the thylakoid membrane proteins of treated tobacco leaves. We compared the responses of the photosynthetic apparatus to *Pst* infection under light and dark conditions.

## Results

### Effects of *Pst* infection on chlorophyll content in the infiltrated area of tobacco leaves

**Fig. 1:**
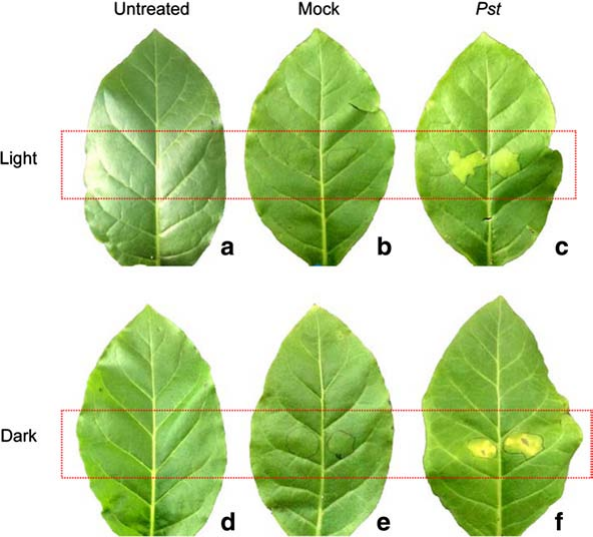
Representative images of tobacco leaf changes following *Pst* infection. Leaves were inoculated with distilled water (mock) or *P. syringae* pv*. tabaci* (*Pst*) for 3 days under light (**a**, **b**, **c**) or dark conditions (**d**, **e**, **f**)

**Fig. 2:**
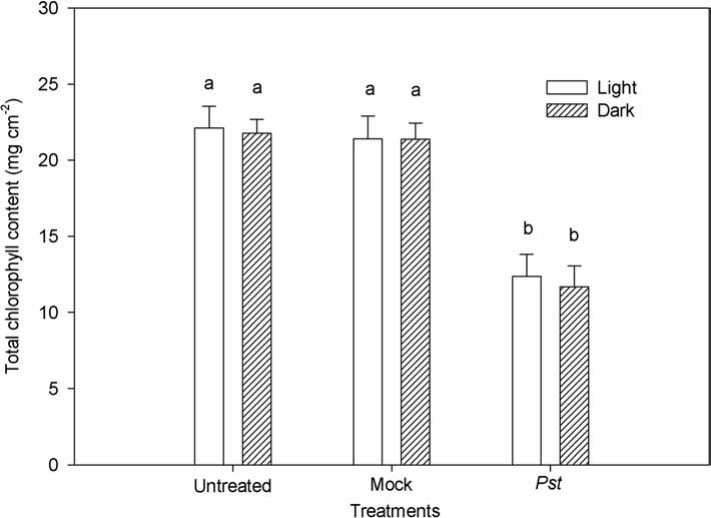
Relative changes in total chlorophyll content at 3 days post *Pst* infection in tobacco leaves. Means ± SE of three replicates are presented. Different letters above the columns indicate significant differences at *P* <0.05 between different treatments

We observed chlorotic lesions in the infiltrated zone at 3 days post infection (dpi), while necrosis was observed at 3 dpi only in leaves treated in the dark. The infiltrated zone of tobacco leaves exhibited obvious wildfire symptoms regardless of whether the leaves were incubated under light or dark conditions (Fig. 1). The total chlorophyll content in infected leaves at 3 dpi was lower than that of untreated leaves (Fig. 2).

### Effects of *Pst* infection on the donor and acceptor sides and the reaction center of PSII in tobacco leaves

**Fig. 3:**
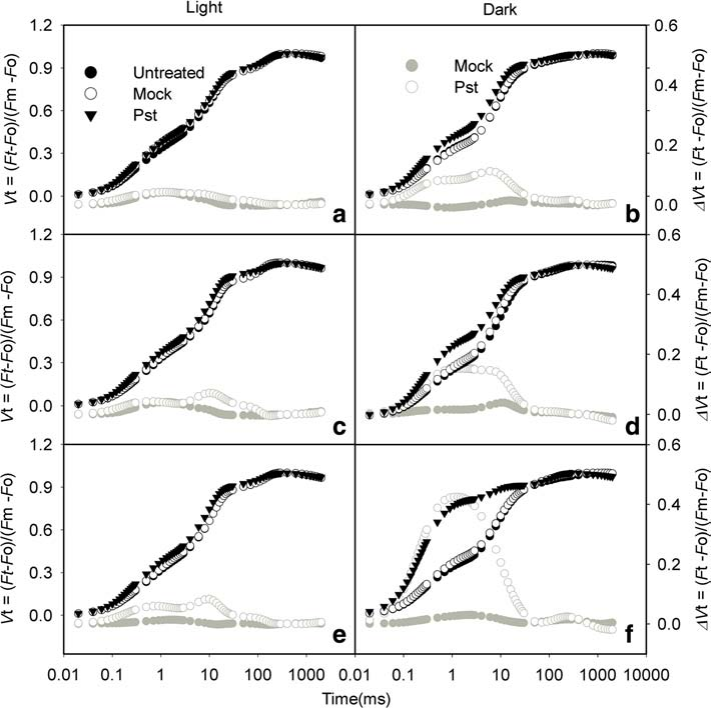
Relative changes in chlorophyll fluorescence induction kinetics during *Pst* inoculation of tobacco leaves. Leaves were inoculated with distilled water (mock) or *P. syringae* pv*. tabaci* (*Pst*) for 1 (**a**, **b**), 2 days (**c**, **d**), or 3 days (**e**, **f**) under light or dark conditions. The K point indicates the K step at about 300 μs and the J point indicates the J step at about 2 ms. *ΔV*_t_ was determined by subtracting the kinetics of the untreated leaves from the kinetics of leaves treated with distilled water or *Pst*. The black symbols correspond to the left y axis and the grey symbols correspond to the right y axis. Every curve is the average of 10 replicates

**Fig. 4:**
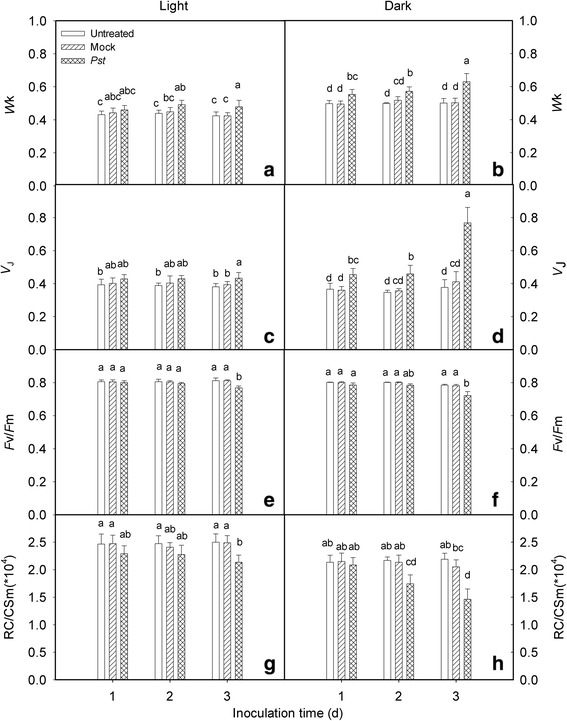
Relative changes in *W*_K_, *V*_J_, *F*_v_/*F*_m_, and RC/CSm after *Pst* infection in tobacco leaves. Chlorophyll *a* fluorescence transients were analyzed with the JIP-test. The *W*_K_ (a, b), *V*_J_ (c, d), *F*_v_/*F*_m_ (e, f), and RC/CSm (g, h) values were calculated after tobacco leaves were inoculated with distilled water (mock) or *P. syringae* pv*. tabaci* (*Pst*) for specific periods under light or dark conditions. Means ± SE of 10 replicates are presented. Different letters above the columns indicate significant differences at *P* <0.05 between different treatments

We used the JIP-test to detect PSII changes in *Pst*-infected tobacco leaves under light and dark conditions. To clarify the effects of *Pst* on PSII, OJIP curves were normalized to the (*F*_m_ − *F*_o_) level. The shape of the OJIP transient changed over time, with the K and J points increasing markedly and the amplitude increasing along with the inoculation time (Fig. 3). The K step (at 300 μs) of the chlorophyll *a* fluorescence transient (quantified as *W*_K_) has been widely used as a specific indicator of oxygen evolving complex (OEC) injury in the photosynthetic apparatus [28, 29]. We observed that *W*_K_ increased after *Pst* infection under light and dark conditions. The increase was more pronounced with increasing time, suggesting that the activity of the donor side of PSII was inhibited and that the OEC was damaged. Compared with that of untreated leaves, *W*_k_ increased by 12.9 and 25.6 % at 3 dpi under light and dark conditions, respectively (Fig. 4a, b). The relative variable fluorescence at the J-step (*V*_J_) represents the subsequent kinetic bottleneck of the electron transport chain, resulting in the momentary maximum accumulation of *Q*_A_^−^ [30, 31]. *V*_J_ is an indicator of the level of closure of PSII reaction centers or the redox state of *Q*_A_ [32]. In this study, compared with untreated leaves, *V*_J_ increased by 13.9 and 103 % in the infiltrated zone at 3 dpi under light and dark conditions, respectively (Fig. 4c, d). Thus, electron transport from *Q*_A_ to *Q*_B_ was severely blocked after *Pst* infection in tobacco leaves. Moreover, inhibition of the K and J steps was more pronounced in the dark, as indicated by the greater increase of the *W*_k_ and *V*_J_ values in the dark during *Pst* inoculation (Fig. 4a-d). The maximum quantum yield of PSII (*F*_v_/*F*_m_) and the density of *Q*_A_^−^ reducing PSII reaction centers per cross section (RC/CSm) values decreased to 94.7 nd 85.4 % of the values of untreated leaves (under light conditions) at 3 dpi, respectively (Fig. 4e, g). The *F*_v_/*F*_m_ and RC/CSm values of treated leaves decreased to 91.9 and 66.8 % of the values of untreated leaves (under dark conditions) at 3 dpi, respectively (Fig. 4f, h).

### Effects of *Pst* infection on PSI complex activity in tobacco leaves

**Fig. 5:**
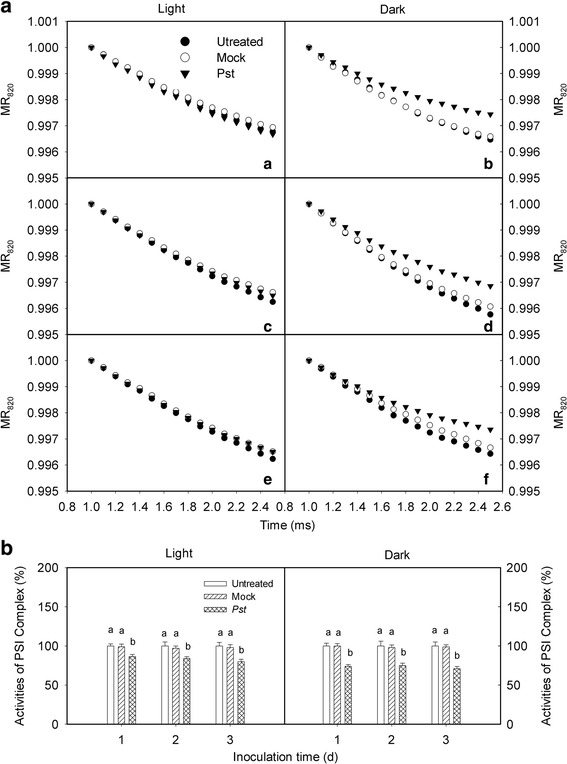
Relative changes in PSI complex activity after *Pst* infection in tobacco leaves. **a**. Modulated reflected signal of 820 nm (MR_820 nm_) was evaluated after leaves had been inoculated with distilled water (mock) or *P. syringae* pv*. tabaci* (*Pst*) for 1 (a, b), 2 (c, d), or 3 days (e, f) under light and dark conditions. The treated leaves were illuminated with red light (2.5 s) and the MR_820nm_ signal changes were simultaneously recorded. The initial MR_820nm_ rate indicates PSI activity. Every curve is the average of 10 replicates. **b**. The PSI complex activity was evaluated after leaves were inoculated with distilled water (mock) or *Pst* for different periods under light (a) and dark (b) conditions. The initial PSI complex activity of untreated tobacco leaves was considered 100 %, while the activities of mock- and *Pst*-treated leaves were calculated as the percentage of activity in untreated leaves. Means ± SE of 10 replicates are presented. Different letters above the columns indicate significant differences at *P* <0.05 between different treatments

We observed considerable differences in PSI activity among treated leaves. The PSI complex activities of treated leaves were 80.0 and 70.8 % of the activity of untreated leaves at 3 dpi under light and dark conditions, respectively (Fig. 5). This indicates that P700 photo-oxidation was rapidly and effectively impaired by *Pst* infection in tobacco leaves under light and dark conditions. Further, the extent of the decrease in PSI activity was greater in the dark (Fig. 5).

### Effects of *Pst* infection on carbon assimilation in tobacco leaves

The net photosynthetic rate (Pn), stomatal conductance (Gs), and carboxylation efficiency (CE) values of treated leaves were 69.3, 17.5, and 21.1 % lower than those of mock controls at 3 dpi, respectively. In contrast, the intercellular CO_2_ concentration (Ci) value of treated leaves was 23.6 % higher than that of mock controls at 3 dpi (Table. 1).

**Table 1:** Relative changes to carbon assimilation parameters at 3 days post *Pst* infection in tobacco leaves

	Pn (μmol m^−2^ s^−1^)	Gs (mmol m^−2^ s^−1^)	Ci (μmol mol^−1^)	CE (μmol m^−2^ s^−1^)
Mock	5.8 ± 0.53a	63 ± 5.29a	225 ± 16.5b	0.0521 ± 0.006a
*Pst*	1.78 ± 0.23b	52 ± 6.08b	278 ± 20.6a	0.0411 ± 0.008b

The changes to net photosynthetic rate (Pn), stomatal conductance (Gs), intercellular CO_2_ concentration (Ci), and carboxylation efficiency (CE) were evaluated. The mean ± SE of four replicates are shown. Different small letters present on the same column indicate significant differences at *P* <0.05 between different treatments

### Relative ROS level changes after *Pst* infection in tobacco leaves

**Fig. 6:**
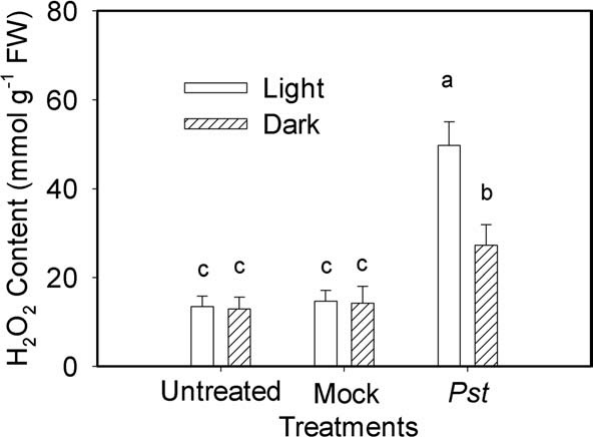
Relative changes in H_2_O_2_ content at 3 days post *Pst* infection in tobacco leaves. Means ± SE of 10 replicates are presented. Different letters above the columns indicate significant differences at *P* <0.05 between different treatments

We evaluated H_2_O_2_ production in the *Pst*-infiltrated zone of tobacco leaves at 3 dpi under light and dark conditions because H_2_O_2_ is the most stable ROS that can be readily measured [33]. The production of H_2_O_2_ was evaluated in the *Pst-*infiltrated zone of tobacco leaves at 3 dpi under light and dark conditions. The H_2_O_2_ content of treated leaves were 269 and 112 % higher than that of untreated controls at 3 dpi under light and dark conditions, respectively (Fig. 6). This implies that an over-accumulation of ROS was induced by *Pst* infection in tobacco leaves under light and, to a lesser extent, dark conditions.

### *Pst*-induced degradation of PsbO, D1, and PsaA proteins in tobacco leaves

**Fig. 7:**
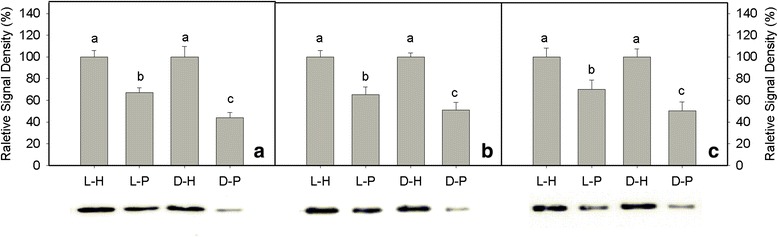
Quantitative image analysis of core protein levels at 3 days post infection in tobacco leaves. PsbO (a), D1 (b), and PsaA (c) protein levels were evaluated. L-H represents leaves infiltrated with distilled water in the light; L-P represents leaves infiltrated with *P. syringae* pv*. tabaci* (*Pst*) in the light; D-H represents leaves infiltrated with distilled water in the dark; and D-P represents leaves infiltrated with *Pst* in the dark. For complete Western blots of PsbO, D1, and PsaA, please see Additional file 1, Additional file 2, Additional file 3. The relative signal density of mock controls was considered 100 %, while the signal density of *Pst* treatments were calculated as the percentage of density in mock controls. Means ± SE of three replicates are presented. Different letters above the columns indicate significant differences at *P* <0.05 between different treatments

The D1 protein pool sizes is representative of the abundance of fully assembled PSII centers as there is one D1 subunit per reaction center. The mature protein is thought to accumulate only when it is integrated into PSII reaction centers. The content of PsbO, D1, and PsaA proteins decreased to 67.0, 65.1 and 70.0 % of the values of water-treated leaves at 3 dpi under light conditions, respectively. The core proteins decreased to 44.1, 51.0 and 50.2 % of the values of water-treated leaves at 3 dpi under dark conditions, respectively (Fig. 7).

## Discussion

We observed lesions consisting of a necrotic center surrounded by chlorotic tissue at 3 dpi in the dark (Fig. 1). Plant pathogens can generally be categorized in three classes (necrotrophs, biotrophs, and hemibiotrophs) on the basis of mechanisms of infection. Biotrophics need living tissue for growth and reproduction. Necrotrophics kill the host tissue during the initial stages of infection and feed on the dead tissue. Hemi-biotrophics exist as biotrophs before switching to a necrotrophic stage [34].

Our study revealed that chlorophyll content decreased considerably during *Pst* inoculation under light and dark conditions (Fig. 2). Chlorophyll degradation has been observed in several plant − pathogen interactions [35, 36]. Kudoh and Sonoike reported that in the early recovery stage after PSI damage, chlorophyll degradation occurred to prevent the absorption of excessive light energy which can otherwise lead to secondary injury of the photosystems [37]. Moreover, Thomas reported that tabtoxinine-β-lactam, a toxin originally described as being from *Pst*, is a dipeptide whose hydrolysis product irreversibly inhibits glutamine synthetase and induces chlorophyll degradation in tobacco leaves [38]. Therefore, the putative tabtoxin activity of *Pst* and the need for photoprotection of the tobacco leaves after PSI damage may have been responsible for the observed chlorophyll degradation.

The reduction of Pn in leaves may have been due to limited CO_2_ diffusion to carboxylation sites as a consequence of decreased stomatal conductance or because of perturbation of enzymatic processes in the Calvin cycle [39]. The decreased Gs and the increased Ci in the *Pst* infiltrated leaves (Table 1) indicated that the decrease in Pn may be the result of a non-stomatal limitation. The decrease in CE (Table 1) indicates that the ribulose 1, 5-bisphosphate carboxylase/oxygenase activity may be inhibited by *Pst* infection, leading to the inhibition of CO_2_ assimilation. Photosynthetic electron transport and carboxylation were both inhibited by *Pst* infection. However, it is unclear whether the effects on PET are the result of inhibition of downstream carboxylation.

The phosphoenolpyruvate carboxylase (EC 4.1.1.31, PEPc) catalyses the irreversible β-carboxylation of phosphoenolpyruvate using HCO_3_^−^ as a substrate in a reaction that yields oxaloacetic acid and inorganic phosphate [40]. Several papers have shown that PEPc activity increased in salt treated *Sorghum bicolor* (a C4 plant), *Hordeum vulgare* (a C3 plant) and *Aleuropus litoralis* (a C3-C4 intermediate plant) [41–43]. The activity of PEPc increased after *Potato virus Y* or *Potato virus A* infection in tobacco leaves [44, 45]. This stimulation of PEPc activity under biotic and abiotic stresses would allow replenishment of the tricarboxylic acid cycle to maintain the activated internal nitrogen metabolism in spite of the reduced photosynthesis rate [46].

The decreases in *F*_v_/*F*_m_ and RC/CSm are conventional indicators of photoinhibition under light conditions [47]. The *F*_v_/*F*_m_ and RC/CSm values decreased considerably as the *Pst* infection progressed (Fig. 4), suggesting that *Pst* infection causes photoinhibition of PSII under light conditions.

Photosystem II is considered to be more vulnerable than PSI when plants encounter stresses because few species have been found in which PSI is more easily photoinhibited than PSII [48, 49]. Photoinhibition of PSI was first reported by Terashima et al. in cucumber plants exposed to low temperature [50]. The PSI activity decreased after *Pst* infection (Fig. 5), indicating that PSI photoinhibition occurred during *Pst* inoculation under light conditions. However, we observed damages to the photosynthetic apparatus during *Pst* inoculation under dark conditions that were similar to the damage caused by photoinhibition induced by light. Therefore, this damage was referred to as “photoinhibition-like damage” which was further indicated by the degradation of PsbO, D1, and PsaA proteins (Fig. 7).

Chloroplasts are the major source of ROS in plant cells. The direct reduction of O_2_ to superoxide by reduced donors associated with PSI occurs during the Mehler reaction [51]*.* The impairment of photosystems inevitably leads to the generation of ROS by the Mehler reaction during *Pst* inoculation (Fig. 6). There are two roles for H_2_O_2_ in plants. At low concentrations, it acts as a messenger molecule involved in signaling related to acclimation and the triggering of defense mechanisms against various stresses [52]. At high concentrations, H_2_O_2_ promotes programmed cell death and oxidative damage [53]. Additionally, H_2_O_2_ can suppress *de novo* D1 protein synthesis by inhibiting elongation factor G [54, 55]. Several reports have suggested that ROS over-production is involved in photoinhibition during various stresses [56, 57]. However, the observed damage to the photosystems was greater and the increase in H_2_O_2_ was much smaller in the dark than in the light (Fig. 6). These results suggest that ROS over-accumulation was not the main reason for the photoinhibition and photoinhibition-like damage induced by *Pst* in tobacco leaves. Additionally, PSI is likely to be attacked by ROS during exposure to stresses, but this attack occurs only if the reduced state of iron-sulfur centers can be maintained, which requires visible light [58]. However, the damage to PSI was greater in the dark, further supporting the viewpoint mentioned above. In accordance with this, Fan et al. indicated that the photoinhibition-like damage of daylily, willow, *euonymus japonicus* and maize was not caused by the over-accumulation of ROS under dark conditions [59].

Counteracting to the negative effects of ROS on the photosynthetic apparatus during photoinhibition, the greater abundance of H_2_O_2_ under light conditions may have led to increased hydroxyl free radical production by the Fenton reaction. The hydroxyl radical may inhibit the pathogen under light conditions [60]. This may be a positive effect of H_2_O_2_ that helped to alleviate photoinhibition and photoinhibition-like damage.

The production of ATP and NADPH during photosynthesis decreases in the dark [61]. The replacement of damaged PSII proteins (primarily the D1 protein) with newly synthesized proteins is an ATP-dependent process [62]. Additionally, the synthesis of the D1 protein of the PSII heterodimer, which is the most rapidly synthesized chloroplast protein, is stimulated by bright light [63]. Therefore, the limited recovery of PSII under dark conditions may be one of the reasons for the greater overall damage observed in the dark during *Pst* inoculation. If a partially repaired PSII in the light minimized the overall damage to the photosystem, it is unclear why the damage to PSI was less extensive in the light than in the dark. The repair of PSI is a very slow process that requires several days or longer. Therefore, the results can not be related to PSI repair. Further studies are needed to clarify this point.

## Conclusions

We evaluated the response of PSI and PSII to *Pst* infection in tobacco leaves under light and dark conditions. The reaction centers and the donor and acceptor sides of the photosystems were all severely damaged, indicating that photoinhibition and photoinhibition-like damage had occurred. We also observed a considerable (net) degradation of PsbO, D1, and PsaA proteins and an over-accumulation of ROS. The accumulated ROS, however, was not the main reason for the photoinhibition and photoinhibition-like damage induced by *Pst* in tobacco leaves. The PsbO, D1, and PsaA proteins appear to be the targets of *Pst* infection under light and dark conditions. Further investigations of photosystem responses may help to identify the main sites of *Pst*-induced damaged in tobacco leaves. This will lead to a better understanding of the mechanisms of plant-pathogen interactions and assist in the breeding of *Pst*-tolerant species.

## Methods

### Plant materials and infiltration with *Pst*

Seeds of tobacco (*Nicotiana tabacum* cv. Longjiang 911, a susceptible cultivar, was kindly supplied by Dr. Jian-Ping Sun, Tobacco Research Institute of Mudanjiang, Mudanjiang, China) were germinated on vermiculite. Forty-five days after germination, the seedlings were transplanted to pots containing a compost-soil substrate to grow in a greenhouse under a natural photoperiod. The two upper fully expanded attached leaves of six to eight weeks old plants were used for experiments.

*Pseudomonas syringae* pv*. tabaci* were grown on solid King’s B agar plates overnight [64], diluted with distilled water to a concentration 10^6^ colony forming units per milliliter. Distilled water (mock) or bacterial suspensions were hand-infiltrated into mesophyll with a needleless syringe on the abaxial side of the leaves. Infiltrating area was about 1 cm^−2^ and measurements were made at a distance of about 0.5 cm from the infiltration area. Following inoculation, the leaves were kept under 14 h light (200 μmol m^−2^ s^−1^) /10 h dark cycles or continuous darkness at 25 °C.

### Measurements of total chlorophyll content in tobacco leaves after *Pst* infection

Leaf total chlorophyll was extracted with 80 % acetone in the dark for 72 h at 4 °C. The extracts were analyzed using a UV-visible spectrophotometer UV-1601 (Shimadzu, Japan) according to the method of Porra (2002) [65].

### Measurement of gas exchange in tobacco leaves after *Pst* infection

The Pn, Gs, and Ci were measured by a CIRAS-3 portable photosynthetic system (PP Systems, USA), which controls the photosynthetic photon flux density at 800 μmol m^−2^ s^−1^, temperature at 25 °C and CO_2_ concentration at 390 μmol mol^−1^ in the leaf chamber. CO_2_ concentration was changed every 3 min in a sequence of 1 600, 1 200, 800, 600, 400, 300, 200, 150, 100 and 0 μmol mol^−1^. Irradiance and CO_2_ concentration were controlled by the automatic control function of the system. CE was calculated according the initial slop of Pn-Ci response curve [66].

### Measurements of the chlorophyll *a* fluorescence transient (OJIP) and PSI activity in tobacco leaves after *Pst* infection

Induction kinetics of prompt fluorescence and the modulated reflected signal of 820 nm (MR_820 nm_) were simultaneously recorded using a Multifunctional Plant Efficiency Analyzer, M-PEA (Hansatech Instrument Ltd., UK) as has been described [67]. All leaves were dark adapted before measurements. Chlorophyll *a* fluorescence transients were analyzed with the JIP-test: *F*_v_/*F*_m_ = 1− (*F*_o_ / *F*_m_); *V*_J_ = (F_2 ms_ − F_o_) / (F_m_ − F_o_); *W*_k_ = (*F*_0.3 ms_ − *F*_o_) / (*F*_2 ms_ − *F*_o_); RC/CSm = φ_Po_ · (*V*_J_ / *M*_o_) • (ABS / CSm), and M_o_ = 4 (*F*_0.3 ms_ − *F*_o_) / (*F*_m_ − *F*_o_); φ_Po_ = *F*_v_/*F*_m_. The MR_820 nm_ signal measured at 820 nm provides information about oxidation state of PSI, including plastocyanin and P700. The induction curve of MR_820 nm_ of the leaves obtained by saturating red light showed a fast oxidation phase and a subsequent reduction phase. The initial slope of the oxidation phase of MR_820 nm_ at the beginning of the saturated red light indicates the capability of P700 to get oxidized, which is used to reflect the activity of PSI [68, 69].

### Detection of H_2_O_2_ generation in tobacco leaves after *Pst* infection

H_2_O_2_ was extracted and determined according to the method of Patterson [70]. Leaf segments (0.5 g) were ground in liquid nitrogen, extracted with 5 ml of 5 % (w / v) trichloroacetic acid and then centrifuged at 16 000 × *g* for 10 min. The supernatant was used for the H_2_O_2_ assay.

### Detection of Psb O, D1, and PsaA proteins in tobacco leaves after *Pst* infection

Thylakoid membranes proteins were detected by Western blot with equal amounts of chlorophyll. Leaves were homogenized in an ice cold isolation buffer [100 mM sucrose, 50 mM Hepes (pH 7.8), 20 mM NaCl, 2 mM EDTA and 2 mM MgCl_2_], then filtered through three layers of pledget. The filtrate was centrifuged at 3000 × *g* for 10 min. The sediments were washed with isolation buffer, re-centrifuged, and then finally suspended in an isolation buffer. The thylakoid membrane proteins were then denatured and separated using 12 % polyacrylamide gradient gel. The denatured proteins in the gel were then electro-blotted to PVDF membranes, probed with antibodies supplied by Fan et al. [59] and then visualized by a chemiluminescence method. Quantitative image analysis of protein levels was performed with Gel-Pro Analyzer 4.0 software.

### Chemicals used in the study

All the compounds used in this study were manufactured by Sigma.

### Statistical analysis

The results presented were the means of at least three independent measurements. Means were compared by analysis of variance and LSD range test at 5 % level of significance.

## Availability of data and materials

All the supporting data are included as additional files.

## Additional files

Additional file 1: Figure S1. PsbO protein level was evaluated at 3 days post infection in tobacco leaves. Lanes from left to right in the picture represent leaves infiltrated with distilled water in the light, leaves infiltrated with *P. syringae* pv*. tabaci* (*Pst*) in the light, leaves infiltrated with distilled water in the dark, and leaves infiltrated with *Pst* in the dark, respectively (PNG 9 kb) Additional file 2: Figure S2. D1 protein level was evaluated at 3 days post infection in tobacco leaves. Lanes from left to right in the picture represent leaves infiltrated with distilled water in the light, leaves infiltrated with *P. syringae* pv*. tabaci* (*Pst*) in the light, leaves infiltrated with distilled water in the dark, and leaves infiltrated with *Pst* in the dark, respectively (PNG 7 kb) Additional file 3: Figure S3. PsaA protein level was evaluated at 3 days post infection in tobacco leaves. Lanes from left to right in the picture represent leaves infiltrated with distilled water in the light, leaves infiltrated with *P. syringae* pv*. tabaci* (*Pst*) in the light, leaves infiltrated with distilled water in the dark, and leaves infiltrated with *Pst* in the dark, respectively (PNG 10 kb)

## Abbreviations

CE: Carboxylation efficiency
Ci: Intercellular CO_2_ concentration
Dpi: Days post infection
*F*_o_: *F*_m_, Initial and maximum fluorescence
*F*_v_*/F*_m_: Maximal quantum yield of PSII
*F*_v_’*/F*_m_’: The quantum yield of open PSII traps
Gs: Stomatal conductance
J: K, Intermediate steps of chlorophyll *a* fluorescence rise between *F*_o_ and *F*_m_
MR_820 nm_: Modulated reflected signal of 820 nm
mSR705: The modified red-edge ratio
NPQ: Nonphotochemical quenching
OEC: Oxygen evolving complex
PEPc: Phosphoenolpyruvate carboxylase
PET: Photosynthesis electron transport
Pn: Net photosynthetic rate
*Pph*: *Pseudomonas* pv*. Phaseolicola*
PSI: Photosystem I
PSII: Photosystem II
*Pst*: *Pseudomonas syringae* pv. *tabaci*
*Pto*: *Pseodomonas syringae* pv. tomatao DC300
*Q*_A_: The primary quinone electron acceptor of PSII
*Q*_B_: The secondary quinone electron acceptor of PSII
RC/CSm: Density of *Q*_A_^−^ reducing PSII reaction centre
ROS: Reactive oxygen species
*V*_J_: The relative variable fluorescence at the J step
*V*_t_: The relative variable fluorescence at the any time
*W*_K_: Normalized relative variable fluorescence at the K step
Φ_PSII_: The actual photochemical efficiency of PSII
