# Supplementary figures and images for: Photoinhibition and photoinhibition-like damage to the photosynthetic apparatus in tobacco leaves induced by pseudomonas syringae pv. Tabaci under light and dark conditions
Source: BMC Plant Biol. 2016 Jan 25;16:29. doi: 10.1186/s12870-016-0723-6 (PMC4727333; doi:10.1186/s12870-016-0723-6)

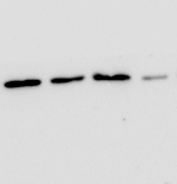

Supplement: Additional file 1: Figure S1. — PsbO protein level was evaluated at 3 days post infection in tobacco leaves. Lanes from left to right in the picture represent leaves infiltrated with distilled water in the light, leaves infiltrated with P. syringae pv. tabaci (Pst) in the light, leaves infiltrated with distilled water in the dark, and leaves infiltrated with Pst in the dark, respectively (PNG 9 kb) [file 12870_2016_723_MOESM1_ESM.png]

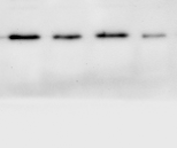

Supplement: Additional file 2: Figure S2. — D1 protein level was evaluated at 3 days post infection in tobacco leaves. Lanes from left to right in the picture represent leaves infiltrated with distilled water in the light, leaves infiltrated with P. syringae pv. tabaci (Pst) in the light, leaves infiltrated with distilled water in the dark, and leaves infiltrated with Pst in the dark, respectively (PNG 7 kb) [file 12870_2016_723_MOESM2_ESM.png]

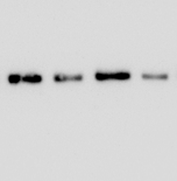

Supplement: Additional file 3: Figure S3. — PsaA protein level was evaluated at 3 days post infection in tobacco leaves. Lanes from left to right in the picture represent leaves infiltrated with distilled water in the light, leaves infiltrated with P. syringae pv. tabaci (Pst) in the light, leaves infiltrated with distilled water in the dark, and leaves infiltrated with Pst in the dark, respectively (PNG 10 kb) [file 12870_2016_723_MOESM3_ESM.png]
